# Supplementary material for: Dissecting human population variation in single-cell responses to SARS-CoV-2
Source: Nature. 2023 Aug 9;621(7977):120–8. doi: 10.1038/s41586-023-06422-9 (PMC10482701; doi:10.1038/s41586-023-06422-9)
Supplement: Supplementary file 1 — Supplementary Notes 1–11 (additional information on the methodology, results and discussion of the main text), Supplementary Figs. 1-10 (additional results) and Supplementary References. [file 41586_2023_6422_MOESM1_ESM.pdf]

nature portfolio

https://doi.org/10.1038/s41586-023-06422-9

Supplementary information

Dissecting human population variation in single-cell responses to SARS-CoV-2

In the format provided by the authors and unedited

Supplementary Information

Dissecting human population variation in single-cell responses to SARS-CoV-2

Yann Aquino, Aurélie Bisiaux, Zhi Li, Mary O'Neill, Javier Mendoza-Revilla, Sarah Hélène Merkling, Gaspard Kerner, Milena Hasan, Valentina Libri, Vincent Bondet, Nikaïa Smith, Camille de Cevins, Mickaël Ménager, Francesca Luca, Roger Pique-Regi, Giovanna Barba-Spaeth, Stefano Pietropaoli, Olivier Schwartz, Geert Leroux-Roels, Cheuk-Kwong Lee, Kathy Leung, Joseph T.K. Wu, Malik Peiris, Roberto Bruzzone, Laurent Abel, Jean-Laurent Casanova, Sophie A. Valkenburg, Darragh Duffy, Etienne Patin, Maxime Rotival & Lluis Quintana-Murci

The **Supplementary Information** file contains: Supplementary Notes 1-11, which contain additional information on the methodology, results and discussion of the main text; Supplementary Figs. 1-10, which present additional results; and Supplementary References

Supplementary Note 1: Single-cell dynamics of the transcriptional responses to SARS-CoV-2 and IAV

To monitor the dynamics of the transcriptional responses to SARS-CoV-2, we performed a kinetic single-cell RNA sequencing experiment on peripheral blood mononuclear cells from a subset of individuals of different ancestry (n = 56 samples, 4 AFB and 4 EUB), following 0, 6 and 24 hours of stimulation with SARS-CoV-2 (BetaCoV/France/GE1973/2020) or IAV (H1N1/PR/8/1934) (Supplementary Fig. 2). After filtering low-quality cells and genetic doublets, we captured 86,177 single-cell transcriptomes, which were assigned to five major cell lineages (myeloid cells, B cells, $CD4^+$ T cells, $CD8^+$ T cells and natural killer (NK) cells) based on per-cluster expression profiles. We found that cell type heterogeneity drives most of the variation in gene expression (40%), while time point and virus exposure accounted for a joint 22% (Supplementary Fig. 2a, b). Cellular composition remained stable over time, except for myeloid cells, which dropped in frequency after 24 hours of IAV stimulation, probably as a result of productive infection and cell death (Supplementary Fig. 2c). Both viruses induced a strong immune response at 6 hours, with 983 differentially expressed genes (DEGs; FDR < 0.01, $|\log_2 FC| > 0.5$) in response to either of the viruses across cell lineages (Supplementary Fig. 2d and Supplementary Table 2a). While IAV productively infected monocytes, we found no evidence for SARS-CoV-2 replication in PBMCs (Supplementary Fig. 2e), consistent with previous findings[1-3]. The strong immune response and higher cell viability observed at 6 hours post-stimulation, together with the presence of IAV-infected myeloid cells, lead us to retain this time point for population-scale analyses.

We observed that transcriptional responses to SARS-CoV-2 and IAV were highly correlated across cell types, with both viruses, especially IAV, strongly inducing interferon-stimulated genes (ISGs) such as *ISG15*, *MX1* or *RSAD2* (Supplementary Fig. 2f). However, monocyte responses were highly heterogeneous, with SARS-CoV-2 inducing a specific transcriptional network enriched in inflammatory-response genes (GO:0006954; odds ratio (OR) > 10.2, p-value < 7.8×10^{-14}) (Supplementary Table 2b, c). Specifically, *IL1B* and *CXCL8* were strongly upregulated in response to SARS-CoV-2 ($\log_2 FC > 2.2$, p-value < 7.1×10^{-20}) but not IAV ($\log_2 FC < 0.01$). Protein assays confirmed the weaker induction of type I and II IFNs by SARS-CoV-2 as well as the specific upregulation of inflammatory cytokines, such as IL-1β, IL-6 or TNF-α (Supplementary Fig. 2g and Supplementary Table 2d). Because monocytes are productively infected by IAV, and not by SARS-CoV-2, we compared the transcriptome of SARS-CoV-2-stimulated monocytes with

that of the fraction of non-infected monocytes in the IAV condition. We observed that the inflammatory profile of SARS-CoV-2-stimulated monocytes remained highly significant (GO:0006954; OR > 10.8, p-value < 5.3×10^{-16}; Supplementary Table 2e). This observation suggests that differences in the sensing of viral particles, rather than infection itself, account for the increased inflammatory response observed for SARS-CoV-2.

Supplementary Note 2: Effects of viral stimulation on cell abundance

To assess the extent to which inferred cell states were comparable across stimulation conditions, we conducted differential abundance analyses by contrasting the number of cells assigned to a given cell type or lineage between stimulated (SARS-CoV-2, IAV) and non-stimulated (NS) conditions (Supplementary Table 3e). We detected significant differences in 46% and 55% of cell types (Bonferroni-adjusted Wilcoxon's signed rank p-value < 0.01) following stimulation with SARS-CoV-2 and IAV, respectively. Yet, for over half of these cell types, the effect of stimulation was very limited (i.e., a change of < 5 cells per sample or < 10% of cells relative to the NS condition). Among the strongest differences, we observed a decrease of up to 62% in the number of $CD14^{+}$ and $CD16^{+}$ monocytes in response to both viruses, as well as an IAV-specific transition of myeloid cells towards infected monocytes, suggestive of stimulation-induced differentiation and cell death upon IAV treatment. Note, however, that because cell types were called separately in each condition, the detected differences in cell abundance could also reflect preferential assignment to specific cell types upon stimulation (i.e., technical differences). For example, the increase that we observe in $CD56^{bright}$ NK cells upon stimulation with both viruses could reflect either the transition of $CD56^{dim}$ NK cells to an activated program where CD56 is upregulated[4], or a technical difference in how NK cells that display an intermediate phenotype are assigned to the $CD56^{bright}$/$CD56^{dim}$ subsets after stimulation.

Supplementary Note 3: Mapping the genetic basis of cell composition

To assess how human genetic factors contribute to population differences in cellular proportions, we mapped the genetic determinants of cell composition at two different levels: (i) considering the number of cells from each of the 5 immune lineages in our setting, relative to the total number of cells in the sample, and (ii) considering, within each major lineage, the number of cells from the different cell types, relative to the total number of cells from the lineage. We found 5 and 13 loci with a genome-wide significant association (p-

value < 5 × 10^{-8}, Supplementary Table 4e) at the lineage and cell type levels, respectively, but only one (rs143488795, closest to the *PTPRF* gene) remained significant after correction for multiple testing (min. *p*-value = 5.1 × 10^{-10}, Supplementary Table 4e). Among the loci identified, 10 displayed marginal evidence of association (*p*-value <10^{-5}) across multiple cell types/conditions. These include the *IGSF21* locus associated with the frequency of MAIT cells across all conditions, the *RBFOX1* locus associated with opposite effects on the percentage of naïve and effector $CD4^+$ T cells across all conditions, or the *LIMCH1* locus associated with changes in the $CD16^+$/$CD14^+$ monocyte ratio specifically upon SARS-CoV-2 stimulation. However, when accounting for the inflation of effect sizes due to multiple testing (winner's curse)[5], the detected loci each explained < 6% of the variance of cellular proportions of cell type concerned. Overall, these observations support the absence of strong genetic effects affecting variation in cell composition in our setting.

Supplementary Note 4: CMV effects on cell composition across populations

Given the virtual lack of CMV^- individuals among Central African donors, the extent to which latent CMV infection affects the specific cell composition of this population cannot be assessed. Nonetheless, mediation analyses can circumvent this limitation by implicitly assuming that the impact of CMV on cellular proportions is the same across populations. Specifically, mediation analyses estimate the CMV-mediated effect on population differences as the product of the estimated effect of CMV on cellular proportions (inferred from Europeans) and the differences in CMV prevalence between populations. To test this assumption, we took advantage of the East Asian samples, where the prevalence of CMV^+ was of 78%. We compared the impact of CMV serostatus on cellular proportions in Europeans and East Asians (Supplementary Fig. 7). We found that the effect of CMV seropositivity on the percentage of memory-like NK cells and $CD8^+$ EMRA T cells is replicated in the East Asian samples (Student's two-sided *t*-test *p*-value < 0.001 for both cell types), with the effect of CMV on cell composition not being significantly different between populations (interaction *p*-value > 0.26 for both cell types). These analyses provide evidence supporting a similar effect of CMV seropositivity on cellular proportions across human populations, thereby validating our mediation analyses.

Supplementary Note 5: Phenotypes of differentiated NK and CD8$^+$ T cells

Given the reported association of CMV infection with severe COVID-19 (ref.[6]), we investigated how the differences in cell composition induced by CMV might alter the leukocyte responses to SARS-CoV-2 stimulation. We found that memory-like NK cells were characterized by a strong exhaustion phenotype: increased basal and stimulated expression of *LAG3* relative to their non-memory counterparts ($\log_2$FC > 2.1, FDR < 2.2×10^{-12}) and decreased induction of major effector cytokines such as *IFNG* and *GZMB* upon viral stimulation ($\log_2$FC between CD56^{dim} and memory-like NK cells > 1.0 after stimulation by SARS-CoV-2, FDR < 1.5×10^{-6}; Supplementary Table 4g). On the other hand, CD8$^+$ EMRA T cells displayed high expression levels of cytotoxicity genes, such as *GZMB, GNLY* and *NKG7*, relative to central and effector memory T cells, while sharing their ability to elicit a strong inflammatory response relative to naïve CD8$^+$ T cells (i.e., genes that display stronger responses to SARS-CoV-2 in CD8$^+$ EMRA T cells, relative to naïve CD8+ T cells, present a 3.4-fold enrichment in inflammatory genes relative to genome-wide expectations, FDR < 9.0×10^{-4}).

Supplementary Note 6: Identification of ancestry-specific eQTLs

To characterize the extent to which the genetic control of immune response variation may be ancestry-specific, we focused on eQTLs and reQTLs that are frequent among individuals of a given genetic ancestry but rarely observed in others, that is, variants with a minor allele frequency (MAF) > 5% in one ancestry and MAF < 1% in others. At the immune lineage level, we identified 973 such ancestry-specific eQTLs, to which another 538 were added at the cell type level (Supplementary Table 5a, b). Likewise, when mapping the genetic basis of the response to viral stimuli, we identified 199 ancestry-specific reQTLs (Supplementary Table 5c, d), leading to a total of 1,662 ancestry-specific (r)eQTLs. Of these, the majority were observed in African-ancestry individuals (n = 1290 *vs.* 208 and 164 in Europeans and East Asians, respectively), likely reflecting the decrease in genetic diversity that occurred in Eurasians following the out-of-Africa event[7].

There was little overlap between ancestry-specific (r)eQTLs and the (r)eQTLs identified in the PBS analyses: 2-7% of high-PBS (r)eQTLs are ancestry-specific *vs.* 1-11% among all (r)eQTLs, (Supplementary Table 7a). While ancestry-specific (r)eQTLs are predominantly observed at low frequency (98% of ancestry-specific eQTLs have a worldwide MAF < 15%), high-PBS (r)eQTLs are enriched in high-frequency variants (94% have a worldwide

MAF > 15%). Nonetheless, when comparing the percentage of (r)eQTLs among ancestry-specific variants between the three populations (Supplementary Table 7d), we found a significant enrichment of East Asian-specific (r)eQTLs in SARS-CoV-2 reQTLs (OR > 4.2, Fisher's exact *p*-value $< 2.3\times10^{-6}$) and myeloid reQTLs (OR > 6.3, Fisher's exact *p*-value $< 6.2\times10^{-7}$), relative to other ancestry-specific (r)eQTLs. This observation, which is consistent with the enrichment of extreme PBS values among SARS-CoV-2 reQTLs in East Asians, provides further support to the notion that genetic adaptation has favoured the differentiation of immune responses to SARS-CoV-2 in individuals of East Asian ancestry.

Supplementary Note 7: Impact of miRNAs on the IFN-mediated response

Given the established role of miR-155 and its complement miR-155* in regulating the expression of IFN-α (ref.[8]), we assessed the impact of the pDC-specific eQTL (rs114273142) detected at the *MIR155HG* gene (i) on IFN-α protein levels measured by SIMOA and (ii) on levels of ISG activity (Methods). We found no significant association between this pDC-specific eQTL and either IFN-α levels or ISG activity (Student's two-sided *t*-test *p*-value > 0.26). Note, however, that the absence of effect in our experimental setting may be explained by time-dependency. Namely, given the expression dynamics of miR-155 and miR-155* in pDCs after stimulation with a TLR7 ligand, and the inverse effects of these miRNAs on type I IFN expression[8], it is tempting to speculate that the effects of *MIR155HG* expression variation on IFN-α levels might be visible at later time points (e.g., > 12 hours post-stimulation) and were thus not captured in our study (i.e., 6 hours post-stimulation).

To test whether other miRNAs could affect IFN-α levels and ISG activity, we focused on a set of 50 miRNAs previously found to be up-regulated in monocytes upon IAV stimulation ($\log_2$FC > 0.2, see Table S2 from ref.[9]), thus being arguably relevant for the myeloid response to viral infection. Overall, through the mapping of *cis*-eQTLs for the miRNA host genes, we detected 17 variants associated with the expression of 8 miRNA host genes (*CYLD*, *TRIM25*, *TNFAIP6*, *NVL*, *C15orf48*, *HLA-B*, *TRRAP* and *MIR155HG*). However, none of these variants were associated with either IFN-α levels or ISG activity (Student's two-sided *t*-test *p*-value > 0.08). Together, these results suggest that eQTLs that alter the expression of miRNA host genes have a limited effect on the variability of IFN-mediated response at 6 hours of stimulation.

Supplementary Note 8: Replication of single-cell eQTLs across studies

We assessed the extent to which the (r)eQTLs detected in this study overlap with those identified by previous single-cell studies of resting and stimulated PBMCs[10-12]. To do so, we compared, in each lineage and condition, the set of genes for which we detected at least one eQTL (eGenes) with eGenes reported in other studies (Supplementary Table 5e, Supplementary Fig. 8). Despite differences across studies in terms of experimental protocol, statistical methodology, sample size, or genetic ancestry, we observed strong enrichments of our basal eQTLs in eGenes detected in two other studies of resting PBMCs[10,11] regardless of the lineage considered (OR > 4.5, Fisher's exact p-value < 1.2×10^{-85}). Furthermore, we found that, depending on the lineage, 51-76% of eGenes detected in our study have been reported in at least another study, this percentage remaining relatively stable when lowering the FDR for eQTL discovery (57-81% of eGenes at an FDR < 10^{-4}). Importantly, our study identified a total of 3,544 genes with previously unreported eQTLs, including 2,658 with high confidence (FDR < 10^{-4}). Focusing on response eQTLs, we compared our results with those of Randolph *et al.*[12], who report single-cell expression data of PBMCs stimulated with influenza A virus (IAV). We also found an enrichment of our IAV reQTLs in genes reported to harbor IAV-specific eQTLs (OR > 3.2, Fisher's exact p-value < 9.4×10^{-4}). Out of the 1,290 eQTLs detected, in the same immune lineage and experimental condition, by both Randolph *et al.* (*lfsr* < 0.1) and our study (FDR < 0.01), > 98% of effect sizes were estimated to act in the same direction, supporting further the high replicability of our eQTL results. However, the general sharing of reQTLs between the two studies remained limited (10-40% of reQTLs depending on the cell type).

Overall, these analyses show that despite significant overlaps in the (r)eQTLs detected across single-cell eQTL studies, the sets of eGenes identified can largely vary according to the population under study, the conditions of stimulation, or the analytical pipeline. This emphasizes the need for future efforts aiming to adopt a standardized processing of the data and to conduct large meta-analysis of single-cell eQTL datasets.

Supplementary Note 9: Effect of cellular composition QTLs on gene expression

The decomposition of population differences in immune response that are mediated by *cis*-genetic effects or by cell composition relies on the assumption that, at each locus considered, genetic variants are independent of cellular composition. This assumption is consistent with

previous reports that the genetic determinants of variation in cellular proportions tend to be highly polygenic[13], such that most variants altering cell composition have a moderate effect (< 6% of the variance in our data, see Supplementary Note 3). To verify that *cis*-genetic regulatory variants are independent from variants affecting cell composition, we sought to exclude a possible scenario where a SNP altering cell type proportions within a specific immune lineage would result in an apparent eQTL at the lineage level (note that eQTLs detected within homogeneous cell types cannot be driven by changes in cellular proportions). We thus tested the association between all lineage-level eQTLs detected in our study and cellular composition, using a marginal *p*-value threshold of $p < 10^{-5}$. At this threshold, we expect >95% power to detect a genetic effect on cell composition, given that for a cell-composition-driven eQTL, its effect on cell composition must be stronger than that on gene expression.

Of the 9,150 *cis*-eQTLs tested, 4 displayed marginal association with cell composition (Student's two-sided *t*-test *p*-value $<10^{-5}$), but none reached genome-wide significance. Furthermore, only 2 of these eQTLs were associated with cellular proportions within the same lineage where the eQTL was detected. Both eQTLs were also observed across multiple lineages, indicating that their effect on gene expression is unlikely to result from changes in cell composition. These analyses strongly support the notion that none of the genetic variants considered in the mediation analyses acts on gene expression through an indirect effect on cell composition.

Supplementary Note 10: Recurrent adaptations targeting type-I IFN-mediated antiviral immunity

Given that our analyses detected multiple signals of local adaptation targeting eQTLs of antiviral effectors of the type-I IFN-mediated immunity, we wondered whether these adaptive events have favored either an increased or decreased antiviral immunity in specific population groups. To test this hypothesis, we focused on a set of 48 well-annotated antiviral effectors[14] and 97 IFN-α responsive genes (25 of which are also antiviral effectors). Of these, 53 genes (18 antiviral effectors and 46 IFN-α responsive genes, with 11 genes belonging to both categories) had an eQTL with evidence of extreme population differentiation (i.e., top 1% population branch statistic (PBS), Supplementary Table 7a, c) or signals of rapid adaptation (max. $|Z| > 3$, Supplementary Table 7b, c). Then, we tested, for each population, whether selection acting on these eQTLs had preferentially increased or decreased the frequency of

alleles associated with higher expression of antiviral effector/IFN-α responsive genes. We found that selection was associated with increased expression of antiviral effector/IFN-α responsive genes for 21, 30 and 13 eQTLs in African (YRI), European (CEU) and East Asian (CHS) populations, respectively, and decreased expression of antiviral effector genes/IFN-α responsive genes for 22, 23, and 32 eQTLs in the YRI, CEU and CHS populations, respectively.

These observations suggest that, in each population, local adaptation had antagonistic effects on the expression of antiviral effectors. In some cases, we observed antagonistic effects of adaptation for multiple eQTLs of the same gene. This is illustrated by the interferon-inducible transmembrane protein 2 (*IFITM2*), where Europeans harbor higher frequencies of both an allele that increases *IFITM2* expression in B cells (rs6421983-T) and an allele that decreases *IFITM2* expression in NK and T cells (rs28521954-T). Finally, focusing on the timing at which adaptation occurred, we found that the adaptive signals at eQTLs of the 53 antiviral effector genes/IFN-α responsive genes were spread over the last 56,000 years in each of the three populations (Supplementary Fig. 10). Together, these results support the notion that repeated events of local adaptation have occurred to maintain a balance between strong antiviral immunity and controlled responses to infection, rather than directional selection favoring increased or decreased antiviral immunity in specific human populations.

Supplementary Note 11: Replication of eQTLs in lung tissue

We investigated the extent to which the eQTLs identified in our model of peripheral immune responses (i.e., PBMCs) could inform other processes that occur during infection by respiratory viruses (i.e., in lung tissues - the primary site of infection for respiratory viruses). To do so, we assessed the fraction of eQTLs we detected in resting or stimulated PBMCs where the eQTL index SNP was associated with gene expression in GTEx lung data[15]. Overall, we found that 38% of our eQTLs were also detected in the lung (Student's two-sided *t*-test p-value < 0.01, same direction effect in the lung and in the immune lineage where the eQTL was identified, Supplementary Table 9b). This percentage was 4.7-fold higher than that of random SNPs matched for distance to the nearest gene and MAF (95% CI: 4.6-5.1, one-sided resampling p-value < 10^{-4}). Furthermore, the percentage of eQTLs shared between PBMCs and lung tissue was higher among eQTLs that are detected across multiple immune

lineages, ranging from 31% for lineage-specific eQTLs to 72% for eQTLs that are shared across the 5 immune lineages (two-sided Fisher's *p*-value = 1.1×10^{-118}).

Furthermore, we found that up to 56% of the eQTLs detected in PBMCs that colocalize with COVID-19 risk loci are also detected in the lung (OR = 2.1, two-sided Fisher's *p*-value = 0.02). A relevant example is the *MUC20* eQTL allele rs2177336-T, which is associated with higher expression of *MUC20* in $CD4^+$ T cells and decreased risk of infection by SARS-CoV-2. That this eQTL is also detected in the lung (Student's two-sided *t*-test *p*-value < 2.3×10^{-16}) suggests that rs2177336-T decreases the risk of SARS-CoV-2 infection through its effect on *MUC20* expression in the respiratory tract, where *MUC20* is most strongly expressed by ciliated epithelial cells (Human Protein Atlas[16]). The remaining 44% of eQTLs detected in basal or stimulated PBMCs that colocalize with COVID-19 risk loci were not detected in the lung tissue, highlighting the relevance of studying responses to SARS-CoV-2 in peripheral immune cells for the dissection of disease mechanisms underlying COVID-19 risk.

Supplementary Figures and Legends

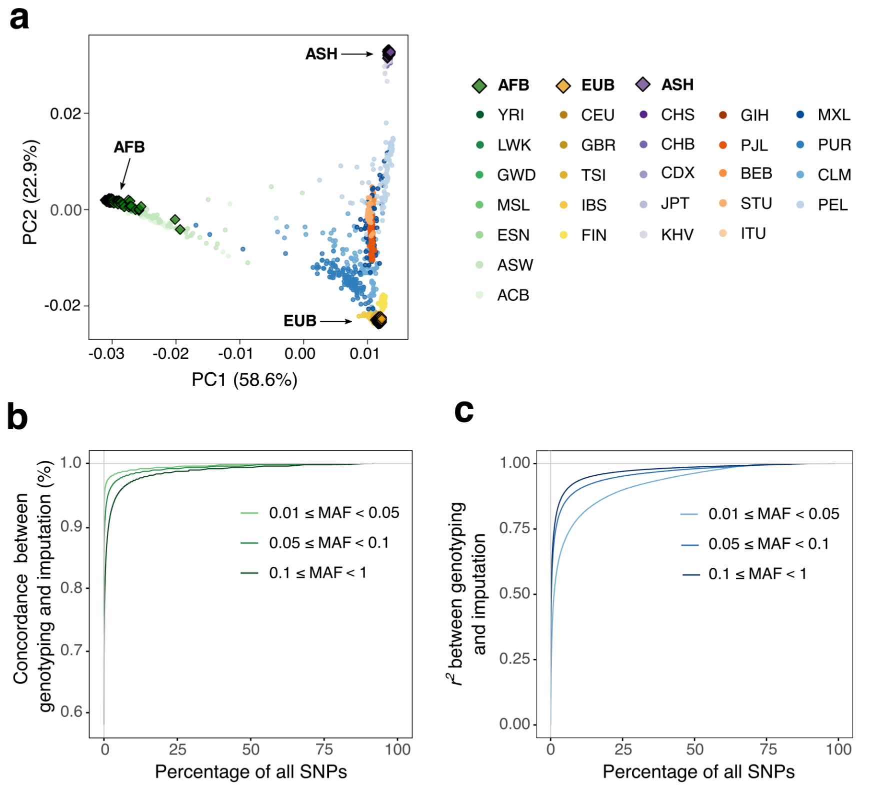

Supplementary Figure 1 | Genetic structure of study populations and SNP imputation. a, Principal component analysis of genotyping data. Each dot corresponds to an individual. Study samples (AFB, EUB, and ASH, in bold typeface) are projected jointly with 1,000 Genomes populations of various ancestries including African (dots colored in green gradient), European (dots colored in yellow gradient), East Asian (dots colored in purple gradient), South Asian (dots colored in orange gradient) and American (dots colored in blue gradient) ancestries. Abbreviations for each individual population can be found in ref.[56]. **b** and **c,** Quality control of genotype imputation. Distribution of genotype accuracy (**b**) and r^2 between genotyped and imputed SNPs (**c**) obtained by 100-fold cross-validation and shown separately for different bins of MAF.

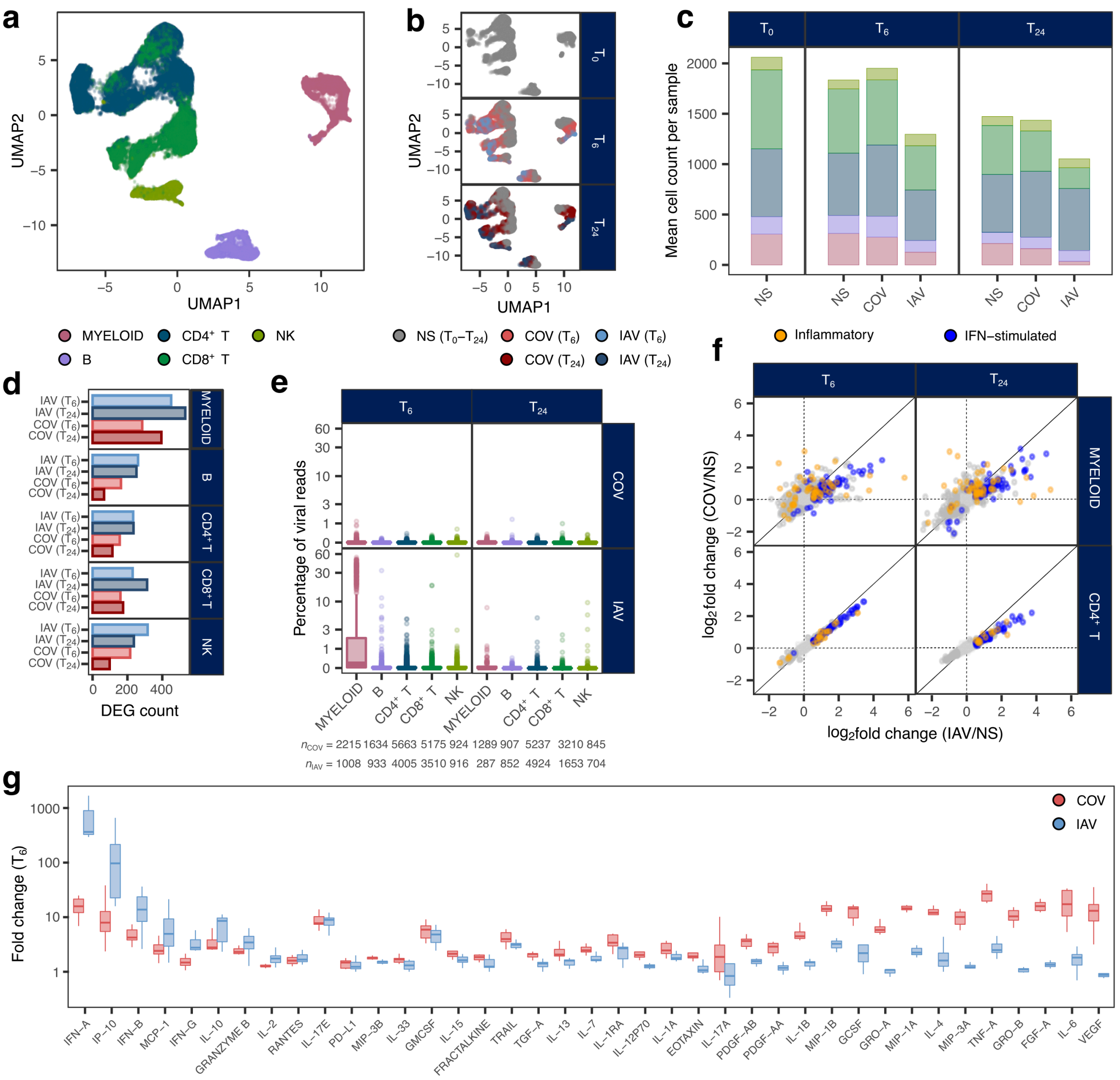

Supplementary Figure 2 | Single-cell kinetics of the immune response to RNA viruses. a, b, Uniform manifold approximation and projection (UMAP) of 86,363 peripheral blood mononuclear cells (PBMCs), mock-stimulated (NS) or stimulated with SARS-CoV-2 (COV) or influenza A virus (IAV) for 0, 6 or 24 hours. **c,** Mean cell type counts per individual, set of stimulation conditions and time point. **d,** Number of differentially expressed genes (DEG; absolute $\log_2$ fold change ($|\log_2 FC|) > 0.5$, FDR < 0.01) after 6 or 24 hours of stimulation with SARS-CoV-2 or IAV relative to non-stimulated controls. **e,** Percentage of reads mapping to the SARS-CoV-2 or IAV genomes per cell after 6 or 24 hours of stimulation, split by major immune lineage. Number of cells for each box from left to right were: COV, T_6: 2,215, 1,634, 5,663, 5,175 and 924; IAV, T_6: 1,008, 933, 4,005, 3,510, and 916; COV, T_{24}: 1,289, 907, 5,237, 3,210 and 845; IAV, T_{24}: 287, 852, 4,924, 1,653 and 704. Cells were collected from 8 unrelated donors, across 40 independent stimulations. **f,** Comparison of inflammatory and interferon-stimulated transcriptional responses of myeloid cells and $CD4^+$ T cells (as an example of a lymphoid cell type) after 6 or 24 hours of stimulation with SARS-CoV-2 or IAV. **g,** Cytokine protein responses to 6 hours of stimulation with SARS-CoV-2 or IAV (n = 8 donors, 3 conditions per donor). In **a, c** and **e,** the colors indicate the immune lineages inferred from single-cell transcriptome data. In **b**, **d** and **g,** the colors indicate the stimulation condition and time post-stimulation. In **e** and **g**, boxplots are defined as follows: middle line,

median; box limits, upper and lower quartiles; whiskers, 1.5× interquartile range; points, outliers.

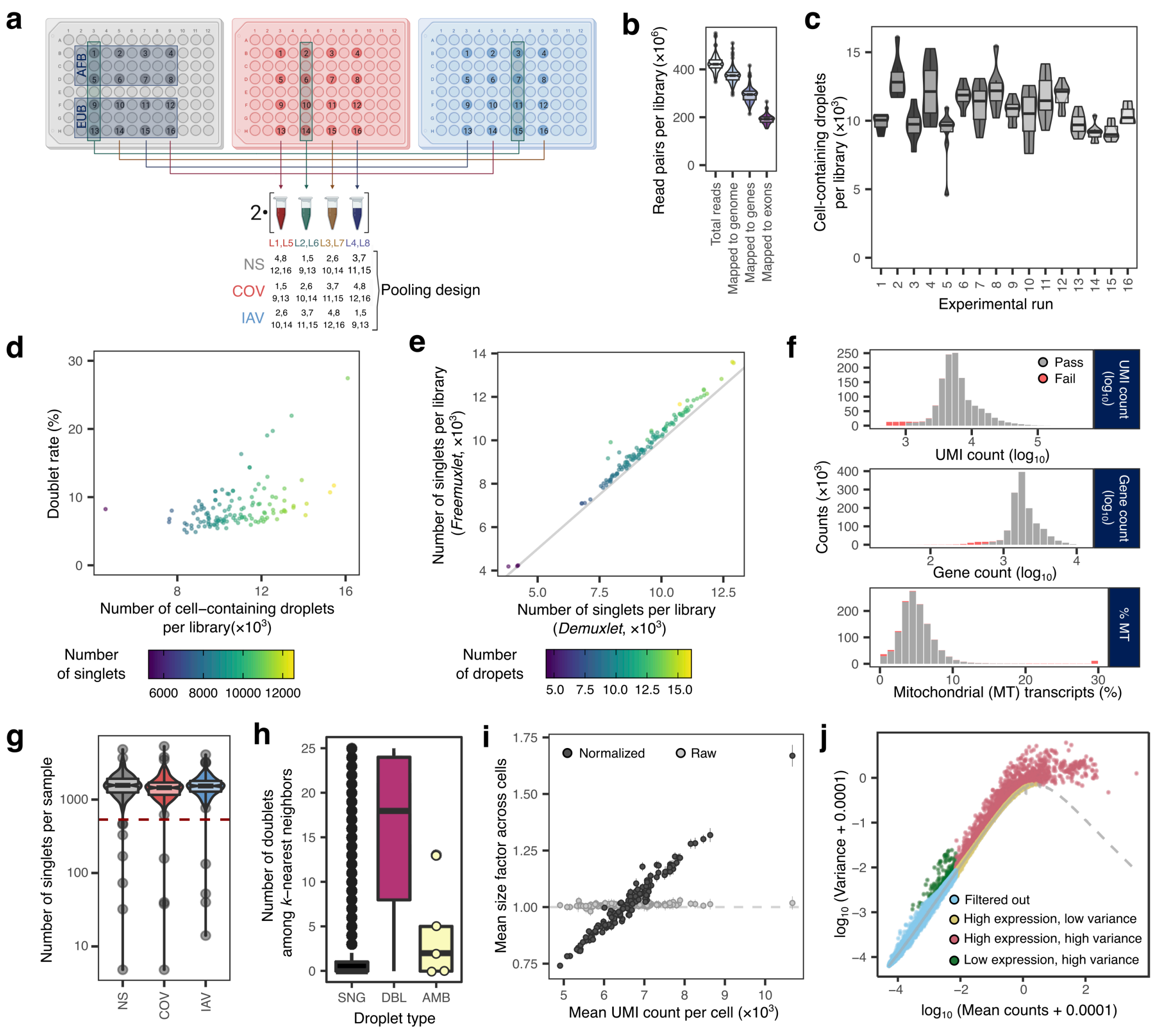

Supplementary Figure 3 | Quality control of single-cell RNA-seq data. a, Experimental design. During each experimental run, PBMCs from 16 individuals (numbered 1 to 16) were processed in three different sets of experimental conditions (colored plates), and the resulting samples were split into four pools of 12 samples (four non-stimulated (NS), four influenza A virus-stimulated (IAV), four SARS-CoV-2-stimulated (COV)). Each pool was then processed on two independent libraries to increase the number of cells per sample (eight pools of 12 samples in total). Figure created using Biorender.com. **b,** Library sequencing depth, and total number of reads aligned either on the whole genome, on the genic regions, or on the coding exons (n = 125 libraries). **c,** Distribution of the number of cell-containing droplets detected per library across the 16 experimental runs performed (n = 8 libraries in all runs except: run 1: n = 5; runs 15 and 16: n = 4). **d,** Percentage of doublets per library as a function of the number of cell-containing droplets detected. Colors reflect the inferred number of singlets in the library. **e,** Number of singlets per library inferred with two independent demultiplexing algorithms: *Demuxlet* (supervised) and *Freemuxlet* (unsupervised). Colors reflect the total number of droplets in the library. **f,** Distribution of cells along quality-control metrics in our data set (i.e., UMI count, gene count, and percentage of mitochondrial reads). Cells that were excluded by our hard-threshold filtering are highlighted in red. **g,** Number of high-quality cells per sample (individual × condition), split by stimulation condition (n = 230 independent biological samples in each condition). Individuals with <500 cells in at least one sample were excluded (dashed red line). **h**, Number of genetic doublets among k nearest neighbors as a

function of the droplet status assigned by *Demuxlet* (SNG: singlet, n = 1,191,463; DBL: doublet, n = 166,858; AMB: ambiguous, n = 5). **i**, Per-library mean of raw and batch-normalized size factors, as a function of the mean number of UMIs per cell in the library (n = 125 libraries). Vertical bars indicate mean ± 2 s.e.m (95% confidence interval of the mean). After normalization with *multiBatchNorm*, size factors successfully capture differences in read depth across libraries. **j**, Filtering of weakly expressed and low-variability genes. For each gene, the variance and mean counts are shown on a log scale (with an offset of 10^{-4}). The dashed line indicates the expected relationship between mean and variance under a Poisson distribution (technical noise). Genes are colored according to their expression levels (highly expressed: mean > 0.01) and estimated biological variance (highly variable: biological variance > 0.001). Genes that are both weakly expressed and of low variability (light blue) are excluded from downstream analyses. In **b, c, g,** and **h**, boxplots are defined as follows: middle line, median; box limits, upper and lower quartiles; whiskers, 1.5× interquartile range; points, outliers.

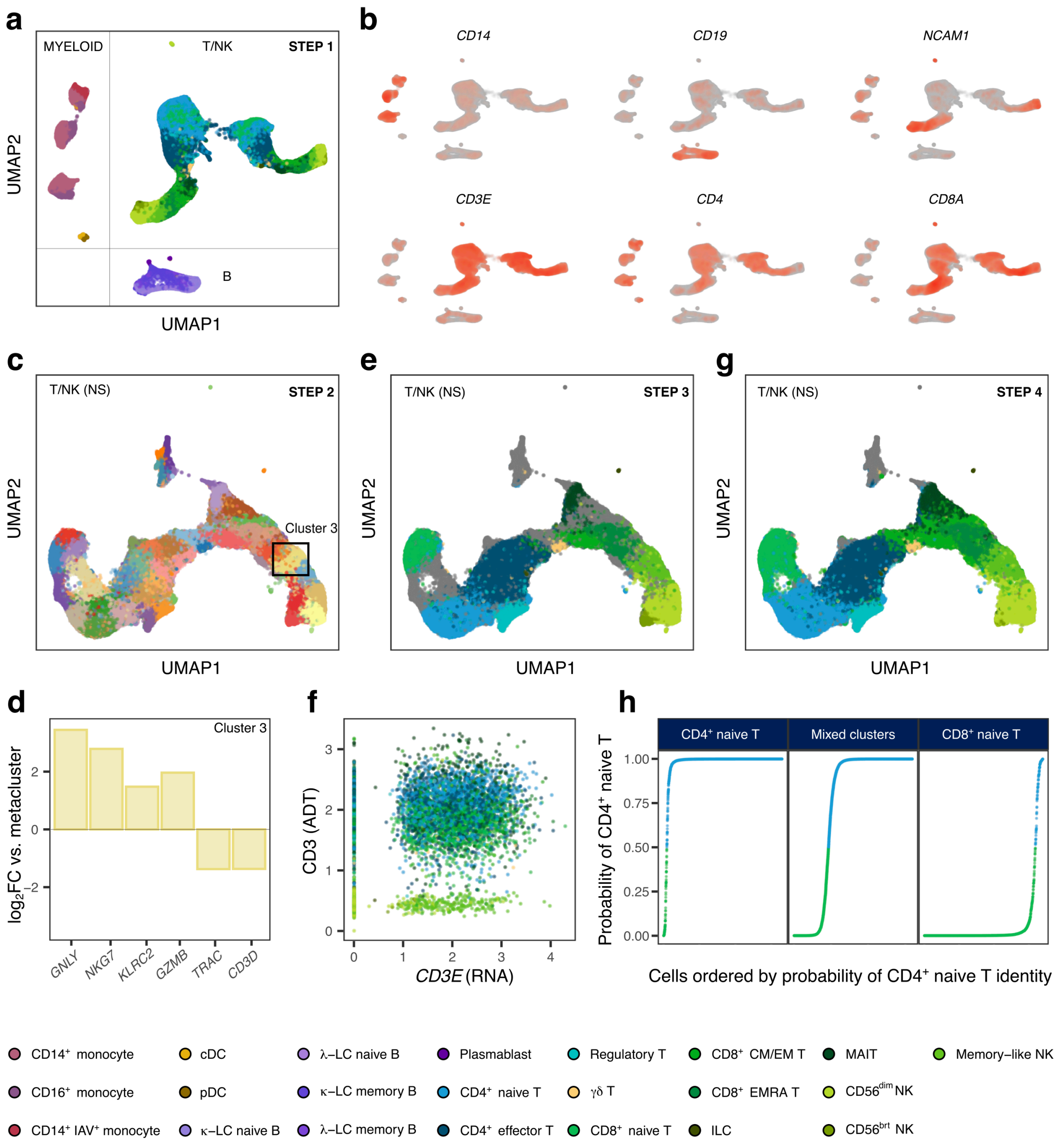

Supplementary Figure 4 | Cell type assignment according to cluster-based transcriptional profiles and surface protein markers. a, Uniform manifold approximation and projection (UMAP) of 1,047,824 PBMCs, either resting (mock-stimulated) or stimulated with SARS-CoV-2 (COV) or influenza A virus (IAV) for 6 hours. **b,** Normalized single-cell RNA UMI count distributions of 6 canonical marker genes. **c,** Graph-based sub-clustering of the non-stimulated T/NK meta-cluster; cluster 3, initially defined as part of a larger cluster of mixed NK and CD8$^+$ T cells, is highlighted. **d,** Log$_2$-fold change difference in expression of markers defining NK cell identity between cluster 3 and the rest of the T/NK metacluster. **e,** Cell type inference based on canonical marker expression in sub-clusters increases the resolution of cell type identities, but some mixed-identity and unidentified clusters remain (gray). **f,** At the transcriptional level, most of the cells in cluster 3 are *CD3E*-positive, and, thus, associated with lymphocyte lineages, but CITE-seq data show that most cluster 3 cells do not express CD3 protein, hence their assignment to the NK lineage. **g,** Cell type inference after CITE-seq-based assignment and resolution of mixed-identity clusters by linear

discriminant analysis (LDA). Unassigned cells (gray) are discarded. **h,** Assignment of cells from mixed-identity clusters, based on previously identified clusters. In this example, LDA models are trained on data from 10,000 confidently identified naive $CD4^+$ and naive $CD8^+$ T cells, making it possible to assign most cells from a mixed cluster to one of the two target identities.

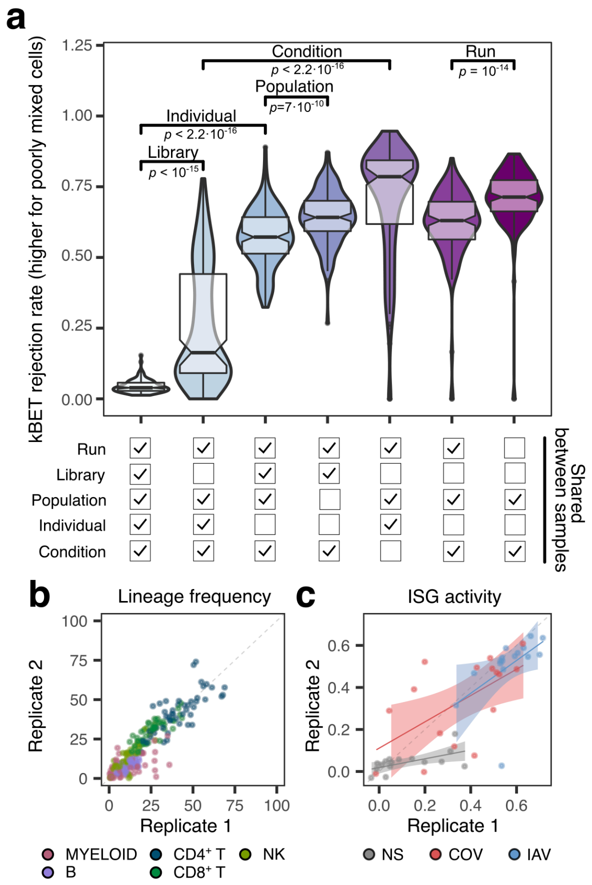

Supplementary Figure 5 | Batch effects and replicability of single-cell experiments. a, Effect of technical and biological variation on the clustering of cells. We quantified kBET rejection rate, which increases when cells from different samples tend to cluster separately, for various comparisons. Sample comparisons were split into seven groups based on the sharing of characteristics among the samples being compared (experimental run, library preparation, individual, population & condition). For each group, we consider a random subset of n=150 pairs of samples (run × library × individual × condition) to reduce computational burden. For each group, violins and boxplots show the distribution of the (middle line: median; box limits: upper and lower quartiles; whiskers: 1.5× interquartile range; points: outliers). For self-comparisons, cells from the same sample were randomly split into two groups before kBET calculation. Comparisons for quantifying the effects of various factors (e.g., run, library preparation, individual, population or stimulation condition) on cell mixing are highlighted. For all comparisons, Wilcoxon's two-sided rank-sum p-value are shown. **b** and **c,** Comparison of estimated lineage proportions (b) and mean ISG expression (c) between replicate samples processed separately across different runs (cells from the same individual thawed on a different day and stimulated in the same conditions). In **c**, for each stimulation condition, lines show the expected ISG activity in replicate 1 given ISG activity in replicate 2; shaded error bands show the 95% CI (mean ± 2 SEM) around this estimate.

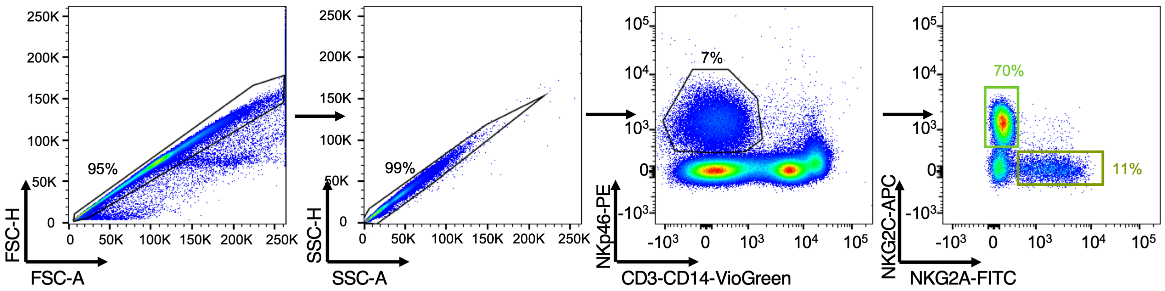

Supplementary Figure 6 | Gating strategy for validation of NK memory subset. Singlets were first selected using FSC-H/FSC-A markers, then with the markers SSC-H/SSC-A. NK cells were then determined as NKp46$^+$/CD3$^+$CD14$^-$Viogreen- cells. From this gate, our subsets of interest were defined as: NKG2C$^+$ cells with NKG2C$^+$/NKG2A$^-$ gate; NKG2A$^+$ cells with NKG2A$^+$/NKG2C$^-$ gate. Finally, a histogram overlay for CD57 marker was made using the cells from the two previous gates (NKG2C$^+$ and NKG2A$^+$ cells), to confirm the phenotypic characteristic of NK memory cells.

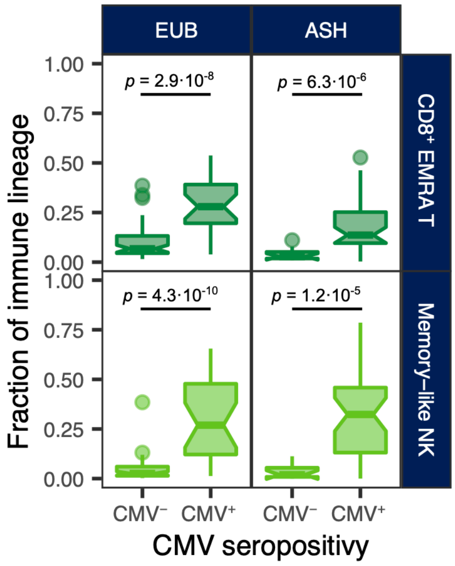

Supplementary Figure 7 | Impact of CMV on cell composition differences among Eurasians. Distribution of $CD8^+$ EMRA T and memory-like NK cell frequencies in West European (EUB) and East Asian (ASH) donors according to cytomegalovirus serostatus ($CMV^{+/-}$). Number of unrelated donors for each box from left to right were: $CD8^+$ EMRA T, EUB: 54 and 25; EMRA T, ASH: 10 and 38; Memory-like NK, EUB: 54 and 25; Memory-like NK: 10 and 38. Wilcoxon's two-sided rank-sum *p*-value < 0.001 shown. Middle line: median; notches: 95% confidence intervals (CI) of median; box limits: upper and lower quartiles; whiskers: 1.5× interquartile range; dots: outliers.

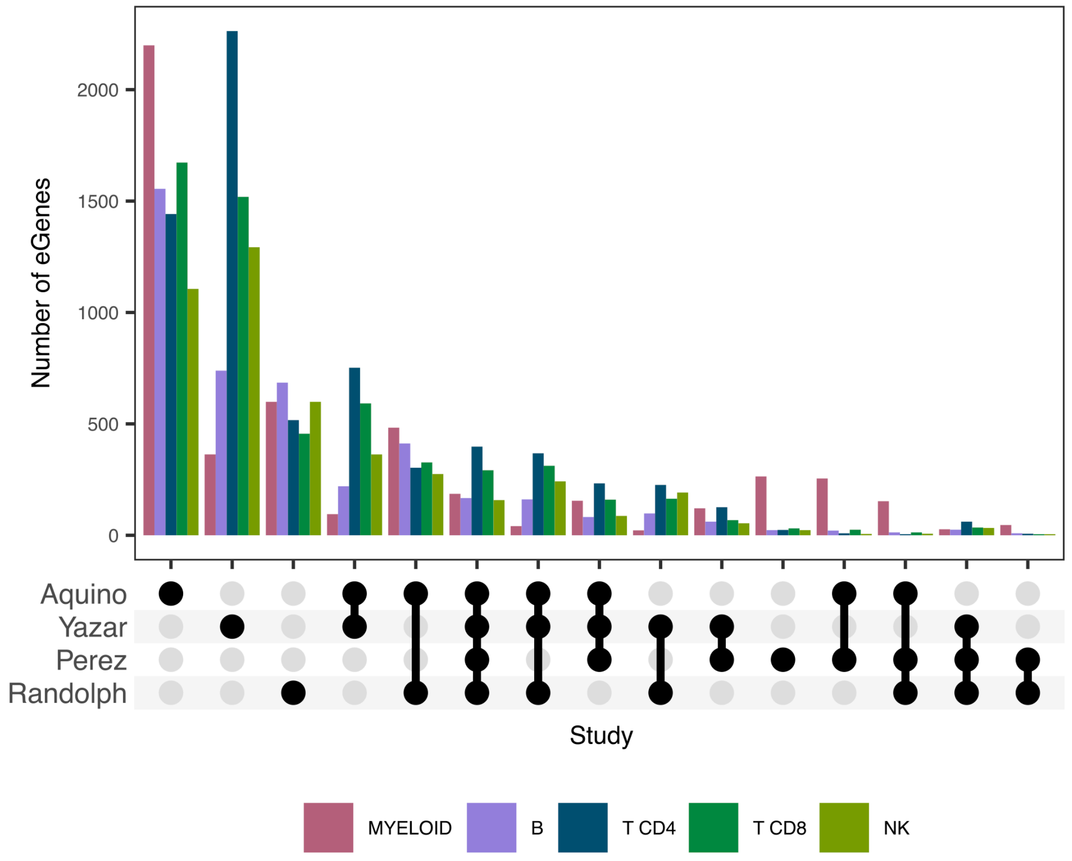

Supplementary Figure 8 | Sharing of eGenes across studies. Identified eGenes from the present study (Aquino *et al.*) are compared with three other single-cell eQTL studies of resting (Yazar *et al.*and Perez *et al.*) or stimulated (Randolph *et al.*) PBMCs. For each possible combination of eQTL studies, the number of shared eGenes is reported for each major immune lineage (coloured bars). Only the 20 combinations with the highest number of eGenes are shown.

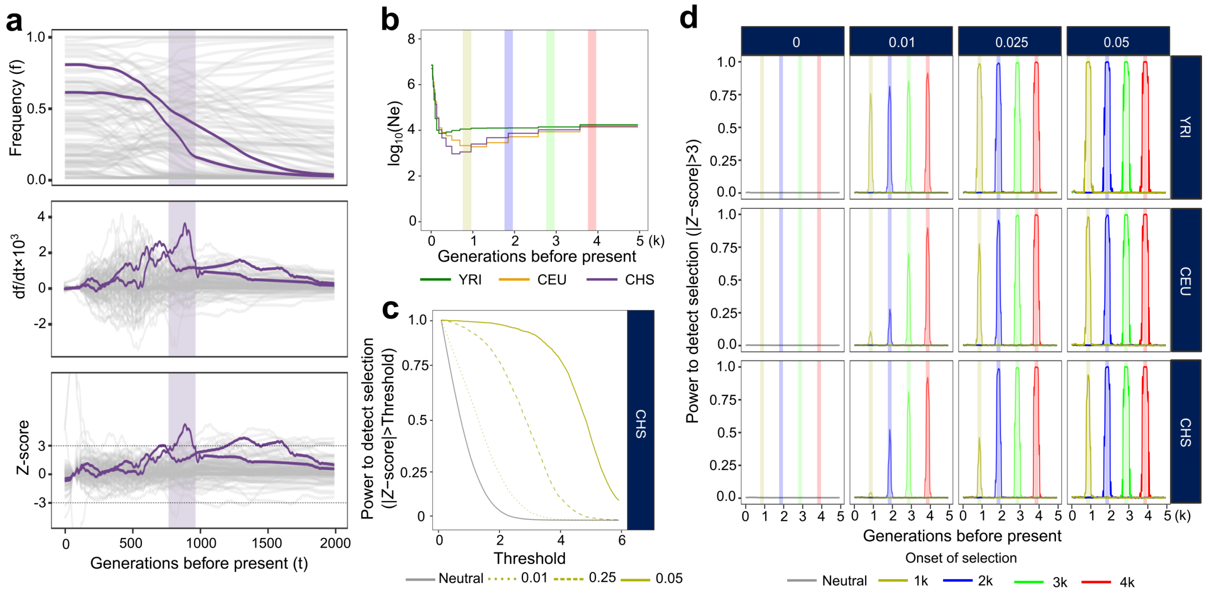

Supplementary Figure 9 | Detecting and timing positive selection signals from allele frequency trajectories. a, Method for estimating the time of onset of selection from allele frequency trajectories. (Top) Allele frequency trajectories in an East Asian population (CHS) across the past 2,000 generations of two SARS-CoV-2 reQTLs (i.e., rs4806787 and rs1028396), affecting the response of *LILRB1* in plasmacytoid dendritic cells and *SIRPA* in $CD14^{+}$ monocytes, respectively. (Middle) Change at each generation (from past to present) of the (smoothed) frequency of the derived allele, normalized for allele frequency. (Bottom) *Z*-score calculated as the normalized derivative, scaled at each generation by the standard deviation of derivatives across all eQTLs. Periods of selection are estimated as the range, in generations, over which the rate of change in the frequency of each allele deviates significantly from expectations under the hypothesis of neutrality (i.e., $|Z\text{-score}| > 3$). (Top to bottom) The corresponding allele frequency trajectories, first derivatives and *Z*-scores for 100 random SNPs sampled from the set of all (r)eQTLs detected in this study are shown in gray. **b,** Effective population size and episodes of positive selection over time used in our simulations. Colored lines indicate effective population size (green: YRI, yellow: CEU, purple: CHS); shaded areas indicate positive selection events. **c,** Type I error and power as a function of *Z*-score threshold. Power and type I error are reported for selection occurring 1000-1200 generations ago, for coefficients of selection ranging from 0.01 to 0.05. **d,** Power to detect positive selection at a *Z*-score threshold of 3, as a function of the time of onset of selection. Lines are colored according to the date on which selection began (gray for neutral).

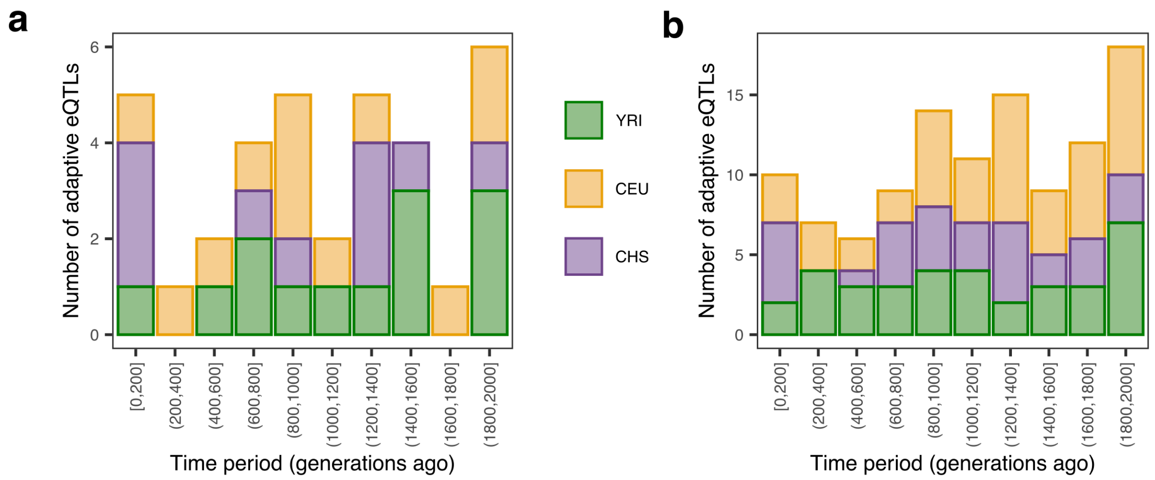

Supplementary Figure 10 | Timing of adaptations targeting antiviral immunity effectors. Distribution of adaptive events, across time periods (i.e., onset of selection) covering the last 2,000 generations (i.e., 56,000 years with a generation time of 28 years), and targeting antiviral immunity in Africans (YRI), Europeans (CEU) and East Asians (CHS). **a**, eQTLs of 18 known antiviral effector genes. **b**, eQTLs of 53 antiviral effector and IFN-α responsive genes.

Supplementary references

1. Ren, X. *et al.* COVID-19 immune features revealed by a large-scale single-cell transcriptome atlas. *Cell* **184**, 1895-1913 e19 (2021).
2. Sungnak, W. *et al.* SARS-CoV-2 entry factors are highly expressed in nasal epithelial cells together with innate immune genes. *Nat Med* **26**, 681-687 (2020).
3. Moustafa, A., Khalel, R.S. & Aziz, R.K. Traces of SARS-CoV-2 RNA in Peripheral Blood Cells of Patients with COVID-19. *OMICS* **25**, 475-483 (2021).
4. Michel, T. *et al.* Human CD56bright NK Cells: An Update. *J Immunol* **196**, 2923-31 (2016).
5. Bigdeli, T.B. *et al.* A simple yet accurate correction for winner's curse can predict signals discovered in much larger genome scans. *Bioinformatics* **32**, 2598-603 (2016).
6. Alanio, C. *et al.* Cytomegalovirus Latent Infection is Associated with an Increased Risk of COVID-19-Related Hospitalization. *J Infect Dis* **226**, 463-473 (2022).
7. Henn, B.M., Cavalli-Sforza, L.L. & Feldman, M.W. The great human expansion. *Proc Natl Acad Sci U S A* **109**, 17758-64 (2012).
8. Zhou, H. *et al.* miR-155 and its star-form partner miR-155* cooperatively regulate type I interferon production by human plasmacytoid dendritic cells. *Blood* **116**, 5885-94 (2010).
9. Rotival, M. *et al.* Population variation in miRNAs and isomiRs and their impact on human immunity to infection. *Genome Biol* **21**, 187 (2020).
10. Perez, R.K. *et al.* Single-cell RNA-seq reveals cell type-specific molecular and genetic associations to lupus. *Science* **376**, eabf1970 (2022).
11. Yazar, S. *et al.* Single-cell eQTL mapping identifies cell type-specific genetic control of autoimmune disease. *Science* **376**, eabf3041 (2022).
12. Randolph, H.E. *et al.* Genetic ancestry effects on the response to viral infection are pervasive but cell type specific. *Science* **374**, 1127-1133 (2021).
13. Vuckovic, D. *et al.* The Polygenic and Monogenic Basis of Blood Traits and Diseases. *Cell* **182**, 1214-1231 e11 (2020).
14. Schoggins, J.W. & Rice, C.M. Interferon-stimulated genes and their antiviral effector functions. *Curr Opin Virol* **1**, 519-25 (2011).
15. GTEx Consortium. The GTEx Consortium atlas of genetic regulatory effects across human tissues. *Science* **369**, 1318-1330 (2020).
16. Papatheodorou, I. *et al.* Expression Atlas update: from tissues to single cells. *Nucleic Acids Res* **48**, D77-D83 (2020).
