# Supplementary material for: Dissecting human population variation in single-cell responses to SARS-CoV-2
Source: Nature. 2023 Aug 9;621(7977):120–8. doi: 10.1038/s41586-023-06422-9 (PMC10482701; doi:10.1038/s41586-023-06422-9)
Supplement: Supplementary file 2 — Reporting Summary [file 41586_2023_6422_MOESM2_ESM.pdf]

nature portfolio

Corresponding author(s): Maxime Rotival, Lluis Quintana-Murci

Last updated by author(s): Jul 3, 2023

# Reporting Summary

Nature Portfolio wishes to improve the reproducibility of the work that we publish. This form provides structure for consistency and transparency in reporting. For further information on Nature Portfolio policies, see our Editorial Policies and the Editorial Policy Checklist.

## Statistics

For all statistical analyses, confirm that the following items are present in the figure legend, table legend, main text, or Methods section.

| n/a | Confirmed | |
|---|---|---|
| ☐ | ☒ | The exact sample size (*n*) for each experimental group/condition, given as a discrete number and unit of measurement |
| ☐ | ☒ | A statement on whether measurements were taken from distinct samples or whether the same sample was measured repeatedly |
| ☐ | ☒ | The statistical test(s) used AND whether they are one- or two-sided<br>*Only common tests should be described solely by name; describe more complex techniques in the Methods section.* |
| ☐ | ☒ | A description of all covariates tested |
| ☐ | ☒ | A description of any assumptions or corrections, such as tests of normality and adjustment for multiple comparisons |
| ☐ | ☒ | A full description of the statistical parameters including central tendency (e.g. means) or other basic estimates (e.g. regression coefficient) AND variation (e.g. standard deviation) or associated estimates of uncertainty (e.g. confidence intervals) |
| ☐ | ☒ | For null hypothesis testing, the test statistic (e.g. *F*, *t*, *r*) with confidence intervals, effect sizes, degrees of freedom and *P* value noted<br>*Give P values as exact values whenever suitable.* |
| ☒ | ☐ | For Bayesian analysis, information on the choice of priors and Markov chain Monte Carlo settings |
| ☒ | ☐ | For hierarchical and complex designs, identification of the appropriate level for tests and full reporting of outcomes |
| ☐ | ☒ | Estimates of effect sizes (e.g. Cohen's *d*, Pearson's *r*), indicating how they were calculated |

*Our web collection on statistics for biologists contains articles on many of the points above.*

## Software and code

Policy information about availability of computer code

| | |
|---|---|
| Data collection | GenomeStudio (v.2011.1), FlowJo software (v10.7.1), STARsolo aligner (v2.7.8a) |
| Data analysis | PLINK (v1.9), GATK (v4.1.2.0), bcftools (v1.16), tabix (v0.2.6), Beagle5.1 (18may20.d20), SHAPEIT4 (v4.2.1), samtools (v1.10), Demuxlet (v0.1), Freemuxlet (v0.1), R (v4.1.0) with the following packages : scran (v1.20.1), SingleCellExperiment (v1.14.1), batchelor (v1.8.1), Seurat (v4.1.1), Harmony (v0.1.0), kBET (v0.99.6), scuttle (v1.2.1), lme4 (v1.1-27.1), sandwich (v2.5-1), lmtest (v0.9-40), care (v1.1.11), fgsea (v1.18.1), MatrixEQTL (v2.3), sva (v3.40.0), SuSiER (v0.11.42), mediation (v4.5.0), and CrossMap (v0.6.3).<br>CLUES (commit n°7371b86, 27 may 2021), Relate (v1.1.8), SliM (v.4.0.1), S-prime (v.07Dec18.5e2), CRF (Sankararaman et al., Nature 2014), coloc (v 5.1.0), S-PrediXcan (v0.6.11), other custom-generated scripts are deposited on GitHub (www.github.com/h-e-g/popCell_SARS-CoV-2). |

For manuscripts utilizing custom algorithms or software that are central to the research but not yet described in published literature, software must be made available to editors and reviewers. We strongly encourage code deposition in a community repository (e.g. GitHub). See the Nature Portfolio guidelines for submitting code & software for further information.

## Data

Policy information about availability of data

All manuscripts must include a data availability statement. This statement should provide the following information, where applicable:

- Accession codes, unique identifiers, or web links for publicly available datasets
- A description of any restrictions on data availability
- For clinical datasets or third party data, please ensure that the statement adheres to our policy

The single-cell RNA sequencing data generated and analyzed in this study have been deposited in the Institut Pasteur data repository, OWEY, which can be accessed at: https://doi.org/10.48802/owey.e4qn-9190. The genome-wide genotyping data generated or used in this study have been deposited in OWEY and can be accessed at https://doi.org/10.48802/owey.pyk2-5w22. Data access and use is restricted to academic research related to the variability of the human immune response. COVID-19 GWAS summary statistics used in the present study can be downloaded from https://www.covid19hg.org/results/r7. Human (1000G data, low [phase 3] and high [NYGC] coverage), archaic (Vindija and Denisova) and ancestral (EPO6) genomes used can be retrieved from ftp://ftp/1000genomes.ebi.ac.uk/vol1/ftp/release20130502 (1000G phase 3), https://www.internationalgenome.org/data-portal/data-collection/30x-grch38 (1000G high coverage), http://cdna.eva.mpg.de/neandertal/Vindija/ (archaic) and ftp://ftp.ensembl.org/pub/release-71/emf/ensembl-compara/epo_6_primate/ (EPO6), respectively. Uniformly processed summary statistics from GTEx lung tissue were downloaded from http://ftp.ebi.ac.uk/pub/databases/spot/eQTL/sumstats/ (GTEx/lung/ge/all: study_id: QTS000015, dataset_id: QTD000271, file: QTD000271.all.tsv.gz).

## Human research participants

Policy information about studies involving human research participants and Sex and Gender in Research.

| | |
|---|---|
| Reporting on sex and gender | The EvoImmunoPop cohort analyzed in this study (EUB and AFB donors) is constituted of self-reported male individuals. This choice, which was originally made in an effort to minimize non-genetic variation and increase power for the mapping of eQTLs, limits the generality of our findings, which are likely to be male-biased. However, this bias is partially balanced by the presence of female individuals among ASH donors (41 self-reported females). In addition, recent work has shown that the genetic basis of gene expression variation is largely shared between males and females (see 10.1126/science.aba3066), suggesting adequate transferability of our results. Self-reported gender was compared with chromosomal sex (inferred based on genotyping data) and was found to be concordant in all but one individual. All analyses including ASH individuals, who include both males and females, were adjusted for chromosomal sex. |
| Population characteristics | The study populations were composed of 80 male donors of self-reported European descent living in Belgium (EUB), 80 male donors of self-reported African descent living in Belgium (AFB), and 71 donors of East Asian descent living in Hong Kong (ASH; 30 males and 41 females). Inclusion of EUB and AFB was restricted to nominally healthy donors between 19 and 50 years of age at the time of sample collection (2012-2013). Inclusion of ASH donors was restricted to nominally healthy donors between 19 and 63 years of age and who were SARS-CoV-2 naive at the time of the sample collection (2020). |
| Recruitment | Recruitment of donors of West European and Central African ancestries was performed at the Center for Vaccinology (CEVAC) of Ghent University Hospital (Ghent, Belgium), based on self-reported ancestry. Recruitment of donors of East Asian ancestry was performed at the School of Public Health of University of Hong Kong (Hong Kong SAR, China). In both cohorts, sampling of related individuals was avoided because relatedness can confound population genetic analyses. We do not anticipate any bias in our results that could be due to this recruitment strategy. |
| Ethics oversight | All donors were sampled after written informed consent had been obtained, and the study was approved by the ethics committee of Ghent University (Belgium, n° B670201214647), the Institutional Review Board of the University of Hong Kong (n° UW 20-132), and the relevant French authorities (CPP, CCITRS and CNIL). This study was also monitored by the Ethics Board of Institut Pasteur (EVOIMMUNOPOP-281297). |

Note that full information on the approval of the study protocol must also be provided in the manuscript.

# Field-specific reporting

Please select the one below that is the best fit for your research. If you are not sure, read the appropriate sections before making your selection.

☒ Life sciences ☐ Behavioural & social sciences ☐ Ecological, evolutionary & environmental sciences

For a reference copy of the document with all sections, see nature.com/documents/nr-reporting-summary-flat.pdf

# Life sciences study design

All studies must disclose on these points even when the disclosure is negative.

| | |
|---|---|
| Sample size | Target sample sizes of 80 individuals per population and stimulation condition, and ~1,500 cells per sample, were determined based on (i) sample size of previous single-cell eQTL studies (Randolph et al. Science, 2021) and (ii) to ensure >80% power for the detection of eQTLs (MAF>5%) with effect sizes higher than 0.2, at a family-wise error rate of 5%, assuming 10 million SNP-gene pairs tested and residual variance in gene expression of 0.2. |

| | |
|---|---|
| Data exclusions | One East Asian donor was excluded due to the presence of low-quality cells upon thawing. Stimulation experiments were performed on the remaining 230 individuals, for a target number of 770 samples (8 individuals × 7 conditions (2 virus × 2 time points + non stimulated × 3 time points) + 222 individuals × 3 conditions + 48 replicates (16 individuals ×3 conditions)), each processed on two separate libraries. One library (L117) failed during the library preparation stage and was thus discarded. Finally, eight East Asian donors were discarded because the number of cells recovered after quality control was too low (< 500 singlets in at least one sample). |
| Replication | The reproducibility of scRNA-seq profiles was evaluated in two ways. First, we processed cells from each sample on two separate 10x Genomics libraries in each run, enabling us to assess technical variability associated with library preparation for each sample. Second, for 16 samples, we performed an additional run, allowing us to evaluate replicability across experiments using the same protocol on samples from the same individual. All attempts at replication were successful (see Supplementary Figure 5). |
| Randomization | We used a balanced design where each experimental run was mostly composed of an approximately equal number of donors of African, European, and East Asian ancestries. Donors were randomly selected within each population. In each experimental run, resting and stimulated cells from 12 different donors were pooled together according to a pre-established scheme (four resting, four SARS-CoV-2 and four IAV samples per library, eight libraries per experimental run) prior to library preparation. cDNA libraries were then pooled and sequenced by groups of eight within each sequencing flow-cell (either pooling libraries from each run, or from 2 different runs). Thus, each flow-cell contained a randomized, balanced set of > 48 samples. Note that within each population (AFB, EUB and ASH) genotypes are randomized by meiotic recombination, which ensures adequate mixing of alleles across batches. |
| Blinding | When performing stimulations, researchers were blinded to the population of origin of the individual. Genotypes and environmental exposures were unknown a priori during data collection, and researchers were blinded to the population of origin of the individual when assessing serologies. Sequencing and quantification of gene expression were performed using automated pipelines and did not take into account the identity of the sample or the population of origin. |

# Reporting for specific materials, systems and methods

We require information from authors about some types of materials, experimental systems and methods used in many studies. Here, indicate whether each material, system or method listed is relevant to your study. If you are not sure if a list item applies to your research, read the appropriate section before selecting a response.

## Materials & experimental systems

| n/a | Involved in the study |
|---|---|
| ☐ | ☒ Antibodies |
| ☒ | ☐ Eukaryotic cell lines |
| ☒ | ☐ Palaeontology and archaeology |
| ☒ | ☐ Animals and other organisms |
| ☒ | ☐ Clinical data |
| ☒ | ☐ Dual use research of concern |

## Methods

| n/a | Involved in the study |
|---|---|
| ☒ | ☐ ChIP-seq |
| ☐ | ☒ Flow cytometry |
| ☒ | ☐ MRI-based neuroimaging |

## Antibodies

Antibodies used

We used the following antibodies for our study :
1) CITE-seq: full description of the antibodies is provided in the Supplementary Table S3B (clone, reference and supplier).
- TotalSeqTM-B 0046 anti-human CD8 (clone SK1, supplier Biolegend, reference: 344757, dilution: 1/50)
- TotalSeqTM-B 0047 anti-human CD56 (NCAM) (clone 5.1H11 , supplier Biolegend, reference: 362561, dilution: 1/50)
- TotalSeqTM-B 0049 anti-human CD3 (clone SK7 , supplier Biolegend, reference: 344853, dilution: 1/50)
- TotalSeqTM-B 0050 anti-human CD19 (clone HIB19 , supplier Biolegend, reference: 302263, dilution: 1/50)
- TotalSeqTM-B 0053 anti-human CD11c (clone S-HCL-3 , supplier Biolegend, reference: 371523, dilution: 1/50)
- TotalSeqTM-B 0063 anti-human CD45RA (clone HI100 , supplier Biolegend, reference: 304161, dilution: 1/50)
- TotalSeqTM-B 0064 anti-human CD123 (clone 6H6 , supplier Biolegend, reference: 306047, dilution: 1/50)
- TotalSeqTM-B 0072 anti-human CD4 (clone RPA-T4 , supplier Biolegend, reference: 300565, dilution: 1/50)
- TotalSeqTM-B 0081 anti-human CD14 (clone M5E2 , supplier Biolegend, reference: 301857, dilution: 1/50)
- TotalSeqTM-B 0083 anti-human CD16 (clone 3G8 , supplier Biolegend, reference: 302063, dilution: 1/50)
- TotalSeqTM-B 0085 anti-human CD25 (clone BC96 , supplier Biolegend, reference: 302647, dilution: 1/50)
- TotalSeqTM-B 0154 anti-human CD27 (clone O323 , supplier Biolegend, reference: 302851, dilution: 1/50)
- TotalSeqTM-B 0159 anti-human HLA-DR (clone L243 , supplier Biolegend, reference: 307661, dilution: 1/50)
- TotalSeqTM-B 0390 anti-human CD127 (IL-7Rα) (clone A019D5 , supplier Biolegend, reference: 351354, dilution: 1/50)
- TotalSeqTM-B 0410 anti-human CD38 (clone HB-7 , supplier Biolegend, reference: 356639, dilution: 1/50)

2) Flow cytometry: full description of the antibodies is provided in the section Flow Cytometry of the Methods (clone and supplier).
- CD3 VioGreen (clone BW264/56, Miltenyi Biotec, dilution: 1/50),
- CD14 V500 (clone M5E2, BD Biosciences, dilution: 1/50),
- CD57 Pacific Blue (clone HNK-1, Biolegend, dilution: 1/20),
- NKp46 PE (clone 9E2/NKp46, BD Biosciences, dilution: 1/10),
- CD16 PerCP-Cy5.5 (clone 3G8, BD Biosciences, dilution: 1/20),
- CD56 APC-Vio770 (clone REA196, Miltenyi Biotec, dilution: 1/50),
- NKG2A FITC (clone REA110, Miltenyi Biotec, dilution: 1/50),
- NKG2C APC (clone REA205, Miltenyi Biotec, dilution: 1/50)

3) SIMOA: full description of the antibodies is provided in the section Supernatants cytokine assays of the Methods (clone and supplier or origin).
- IFN-α capture antibody (clone 8H1, supplier: Evitria, Switzerland, origin: APS1/APECED patient, concentration: 0.3 mg/mL)
- IFN-α detector antibody (clone 12H5 ,supplier: Evitria, Switzerland, origin: APS1/APECED patient, concentration: 0.3 µg/mL)
- IFN-γ capture antibody (clone MD-1, supplier: BioLegend, concentration: 0.3 mg/mL)
- IFN-γ detector antibody (clone MAB285, supplier: R&D Systems, concentration: 0.3 µg/mL)
- IFN-β capture antibody (clone 710322-9 IgG1 kappa, supplier: PBL Assay Science, origin mouse monoclonal antibody, concentration: 0.3 mg/mL)
- IFN-γ detector antibody (710323-9 IgG1 kappa, supplier: PBL Assay Science, origin mouse monoclonal antibody, concentration: 0.3 µg/mL)

Validation

Validation of commercial antibodies was done on a regular internal quality control for each lot by the manufacturer.
Flow cytometry:
Miltenyi Biotech
https://www.miltenyibiotec.com/upload/assets/dataSheet_p42150_eng_GBR.pdf
https://www.miltenyibiotec.com/upload/assets/IM0022906.PDF
https://www.miltenyibiotec.com/upload/assets/dataSheet_p42217_eng_GBR.pdf
https://www.miltenyibiotec.com/upload/assets/dataSheet_p68857_eng_GBR.pdf
BD Biosciences
https://www.bdbiosciences.com/content/bdb/paths/generate-tds-document.us.561391.pdf
https://www.bdbiosciences.com/content/bdb/paths/generate-tds-document.us.562101.pdf
https://www.bdbiosciences.com/content/bdb/paths/generate-tds-document.us.560717.pdf
Biolegend
https://d1spbj2x7qk4bg.cloudfront.net/en-us/products/pacific-blue-anti-human-cd57-antibody-8827?pdf=true&displayInline=true&leftRightMargin=15&topBottomMargin=15&filename=Pacific%20Blue%E2%84%A2%20anti-human%20CD57%20Antibody.pdf&v=20220914123035

CITE-seq:
Biolegend
https://d1spbj2x7qk4bg.cloudfront.net/en-us/products/totalseq-b0046-anti-human-cd8-antibody-18042?pdf=true&displayInline=true&leftRightMargin=15&topBottomMargin=15&filename=TotalSeq%E2%84%A2-B0046%20anti-human%20CD8%20Antibody.pdf&v=20220902063018
https://d1spbj2x7qk4bg.cloudfront.net/en-us/products/totalseq-b0047-anti-human-cd56-ncam-antibody-18156?pdf=true&displayInline=true&leftRightMargin=15&topBottomMargin=15&filename=TotalSeq%E2%84%A2-B0047%20anti-human%20CD56%20(NCAM)%20Antibody.pdf&v=20210121043158
https://d1spbj2x7qk4bg.cloudfront.net/en-us/products/totalseq-b0049-anti-human-cd3-antibody-19288?pdf=true&displayInline=true&leftRightMargin=15&topBottomMargin=15&filename=TotalSeq%E2%84%A2-B0049%20anti-human%20CD3%20Antibody.pdf&v=20220907063026
https://d1spbj2x7qk4bg.cloudfront.net/en-us/products/totalseq-b0050-anti-human-cd19-antibody-16831?pdf=true&displayInline=true&leftRightMargin=15&topBottomMargin=15&filename=TotalSeq%E2%84%A2-B0050%20anti-human%20CD19%20Antibody.pdf&v=20221026111349
https://d1spbj2x7qk4bg.cloudfront.net/en-us/products/totalseq-b0053-anti-human-cd11c-antibody-18043?pdf=true&displayInline=true&leftRightMargin=15&topBottomMargin=15&filename=TotalSeq%E2%84%A2-B0053%20anti-human%20CD11c%20Antibody.pdf&v=20220820063106
https://d1spbj2x7qk4bg.cloudfront.net/en-us/products/totalseq-b0063-anti-human-cd45ra-antibody-16850?pdf=true&displayInline=true&leftRightMargin=15&topBottomMargin=15&filename=TotalSeq%E2%84%A2-B0063%20anti-human%20CD45RA%20Antibody.pdf&v=20220820063106
https://d1spbj2x7qk4bg.cloudfront.net/en-us/products/totalseq-b0064-anti-human-cd123-antibody-18968?pdf=true&displayInline=true&leftRightMargin=15&topBottomMargin=15&filename=TotalSeq%E2%84%A2-B0064%20anti-human%20CD123%20Antibody.pdf&v=20220830085839
https://d1spbj2x7qk4bg.cloudfront.net/en-us/products/totalseq-b0072-anti-human-cd4-antibody-16820?pdf=true&displayInline=true&leftRightMargin=15&topBottomMargin=15&filename=TotalSeq%E2%84%A2-B0072%20anti-human%20CD4%20Antibody.pdf&v=20220824063016
https://d1spbj2x7qk4bg.cloudfront.net/en-us/products/totalseq-b0081-anti-human-cd14-antibody-16827?pdf=true&displayInline=true&leftRightMargin=15&topBottomMargin=15&filename=TotalSeq%E2%84%A2-B0081%20anti-human%20CD14%20Antibody.pdf&v=20220817071325
https://www.biolegend.com/en-us/products/totalseq-b0083-anti-human-cd16-antibody-16829
https://d1spbj2x7qk4bg.cloudfront.net/en-us/products/totalseq-b0085-anti-human-cd25-antibody-16836?pdf=true&displayInline=true&leftRightMargin=15&topBottomMargin=15&filename=TotalSeq%E2%84%A2-B0085%20anti-human%20CD25%20Antibody.pdf&v=20220820063106
https://d1spbj2x7qk4bg.cloudfront.net/en-us/products/totalseq-b0154-anti-human-cd27-antibody-16839?pdf=true&displayInline=true&leftRightMargin=15&topBottomMargin=15&filename=TotalSeq%E2%84%A2-B0154%20anti-human%20CD27%20Antibody.pdf&v=20220820063106
https://d1spbj2x7qk4bg.cloudfront.net/en-us/products/totalseq-b0159-anti-human-hla-dr-antibody-16879?pdf=true&displayInline=true&leftRightMargin=15&topBottomMargin=15&filename=TotalSeq%E2%84%A2-B0159%20anti-human%20HLA-DR%20Antibody.pdf&v=20220830045305
https://d1spbj2x7qk4bg.cloudfront.net/en-us/products/totalseq-b0390-anti-human-cd127-il-7ralpha-antibody-16859?pdf=true&displayInline=true&leftRightMargin=15&topBottomMargin=15&filename=TotalSeq%E2%84%A2-B0390%20anti-human%20CD127%20(IL-7R%CE%B1)%20Antibody.pdf&v=20220820063106
https://d1spbj2x7qk4bg.cloudfront.net/en-us/products/totalseq-b0410-anti-human-cd38-antibody-18086?pdf=true&displayInline=true&leftRightMargin=15&topBottomMargin=15&filename=TotalSeq%E2%84%A2-B0410%20anti-human%20CD38%20Antibody.pdf&v=20220820063106

Simoa:
Validation of the antibodies used for the Simoa assays has been previously described and published in the following articles: Rodero et al., Detection of interferon alpha protein reveals differential levels and cellular sources in disease. J. Exp. Med. 214 (2017); Hadjadj et al., Impaired type I interferon activity and inflammatory responses in severe COVID-19 patients. Science 369 (2020).

## Flow Cytometry

### Plots

Confirm that:

☒ The axis labels state the marker and fluorochrome used (e.g. CD4-FITC).

☒ The axis scales are clearly visible. Include numbers along axes only for bottom left plot of group (a 'group' is an analysis of identical markers).

☒ All plots are contour plots with outliers or pseudocolor plots.

☒ A numerical value for number of cells or percentage (with statistics) is provided.

### Methodology

| | |
|---|---|
| Sample preparation | Frozen PBMCs from three AFB (CMV+) and six EUB (3 CMV+, 3 CMV-) donors were thawed, centrifuged, counted and allowed to rest overnight. For each donor, 1E6 cells were resuspended in PBS supplemented with 2% FBS and incubated with human Fc blocking solution (BD Biosciences) for 10 minutes at 4°C. Cells were then stained with the following antibodies for 30 minutes at 4°C: CD3 VioGreen (Miltenyi Biotec), CD14 V500 (BD Biosciences), CD57 Pacific Blue (Biolegend), NKp46 PE (BD Biosciences), CD16 PerCP-Cy5.5 (BD Biosciences), CD56 APC-Vio770 (Miltenyi Biotec), NKG2A FITC (BioLegend), NKG2C APC (BioLegend). |
| Instrument | Samples were acquired on a MACSQuant 10 cytometer (Miltenyi, S/N 2428). |
| Software | Data were analyzed using FlowJo v10.7.1. |
| Cell population abundance | Between 0.3x1E6 and 0.5x1E6 PBMCs per donor were acquired on the cytometer. The percentage of NKG2C+ and NKG2A+ cells were determined following the gating strategy described below. |
| Gating strategy | Singlets were first selected using FSC-H/FSC-A markers, then with the markers SSC-H/SSC-A. NK cells were then determined as NKp46+/CD3+CD14-Viogreen- cells. From this gate, our subsets of interest were defined as: NKG2C+ cells with NKG2C+/ NKG2A- gate; NKG2A+ cells with NKG2A+/NKG2C- gate. Finally, a histogram overlay for CD57 marker was made using the cells from the two previous gates (NKG2C+ and NKG2A+ cells), to confirm the phenotypic characteristic of NK memory cells. |

☒ Tick this box to confirm that a figure exemplifying the gating strategy is provided in the Supplementary Information.
